# Nudge for governmental change in health policy

**DOI:** 10.3389/fpubh.2026.1739353

**Published:** 2026-01-30

**Authors:** Hatem H. Alsaqqa

**Affiliations:** ^1^Public Health Department and Deanship of Scientific Research, Al-Quds University, Jerusalem, Palestine; ^2^Palestinian Ministry of Health, Gaza, Palestine

**Keywords:** behavior, change, health, nudge, policy

## Abstract

Legislation, regulation, and information supply are classic policy tools used to influence behavior. Policies that ‘nudge’ people in specific directions have recently attracted the attention of policy analysts, based on the growing realization that context has a significant and mostly automatic impact on behavior. This perspective article examines the health behavior change and explores the theoretical underpinnings of why nudges might be effective. Governmental behavior change policies and interventions in the healthcare industry have historically tended to concentrate on educating people, changing their perspective on their behavior, or offering alternative legal incentives that alter the consequences of their actions. Therefore, choice architecture interventions seek to direct people toward personally and socially desirable behavior without the use of education or substantial incentives. They do this by creating settings and incorporating people’s limitations when making decisions in order to support the evaluation and comparison of available choice alternatives, facilitate access to information that is relevant to decisions, or reinforce actions that have already been formed. Researchers in health policy implementation must continue to analyze the impact of nudges by providing access to health data and facilitating ongoing evidence-based evaluation of nudge-based health policies, even though they have successfully influenced health behavior in some areas. Last but not least, multifaceted and comprehensive approaches to health policy that combine both conventional policies and behaviorally informed solutions are the only way to effectively address the multiple and complicated health issues facing our countries.

## Background

Changing a variety of behaviors at scale across communities, such as how we travel, what we eat and drink, and how we keep our houses at a pleasant temperature, is essential to improving population health. In order to achieve these changes, interventions must be carried out at scale, meaning they must have the capacity to reach every member of a population as defined by geography, such as a nation, region, or city. Interventions that change the settings or locations where the target behavior takes place fall under this category (1).

It can be challenging to alter routine or habitual behaviors, like our eating habits or our travel habits, and there is no one method that consistently produces positive results. It almost always takes several interventions to achieve long-lasting change across communities (2). Nudges have drawn more attention due to their possible application in helping people modify their behavior (3–5). When time is limited, nudges can be used to target automatic cognitive processes without requiring many resources (e.g., primary care setting).

However, research indicates that a range of nudges, including public pledges and feedback from societal norms, may improve for example, appropriate prescription of antibiotics (5). As a result of these positive outcomes, various health systems are now looking into ways to increase the sustainability and reach of nudge interventions. Therefore, among other things, a nudge functions without (i) prohibiting or adding any options for rationally relevant choices, (ii) altering incentives as measured by time, trouble, social sanctions, economics, and so on, or (iii) supplying factual information and logical arguments (6).

Researchers brought to public and policymakers’ attention another set of interventions with the potential to change behavior at scale, so-called nudge or choice architecture interventions. These involve altering some aspect of physical, social or other types of habitual factors to make one behavior more likely than another. However, Thaler and Sunstein distinguished nudging from other comparable interventions by placing it within a libertarian-paternalist political philosophy. They contended that their approach upheld the freedom to act as one pleases (2).

Moreover, for a long time, governments have had strong tools at their discretion to affect population health. These tools can be used both “upstream,” to address some of the root causes of poor health, and downstream, to address issues that arise when adverse health behaviors are already evident. Legislation backed by enforcement actions, such as those that prohibit dangerous substances or control what enters our food supply, will be among them. Fiscal measures have historically been used to raise the cost of alcohol and cigarettes, and less frequently to lower the cost of goods and activities that promote health (7).

Interventions that alter the default for available options, the variety of options, the arrangement of options, and the size of portions, packages, or tableware are commonly included in assessments of their effectiveness, despite the fact that there is no widely recognized typology of interventions that fall under the general heading of nudges (8, 9). The effectiveness of these and other nudges depends on a number of factors, including the population, the behavior being altered, the environment in which the intervention occurs, the nature and extent of the intervention, and other factors.

Furthermore, to affect change across populations, any intervention that has been consistently shown to be beneficial in trials must be scalable (10). Additionally, it must be executed in a way that can reach all members of the population, as well as in national or subnational government programs, public agencies, and private sector organizations. These could be accomplished by a voluntary action, as part of a set of rules, or as required by law (2).

Given the limited public resources, the idea has drawn the interest of governments at all levels who wish to change population-level behaviors by concentrating either directly or indirectly on the physical and social environment (11). At a time when individualization is popular and the one-size-fits-all strategy is being rejected more and more, this is appealingly coupled with the upholding of one’s sense of choice (12).

Since behavior change is the key to making most of these interventions nudge interventions, the WHO speculated that by 2030, investing in practical and cost-effective interventions to prevent and control non-communicable diseases in middle-income countries could yield a sevenfold return. These interventions could deter unhealthy physical activity, manage diabetes and cardiovascular diseases, and reduce alcohol and tobacco use (13). Nonetheless, three key characteristics of a nudge are that it (1) does not compel individuals to adopt a specific behavior, (2) protects their freedom of choice, and (3) does not provide significant financial rewards (14).

The governance lens presented in this perspective article suggests that choice architecture nudges are a useful tool for promoting desirable health behaviors and enhancing policy adherence. It makes clear how behavioral science can be used to inform legislative and regulatory actions that promote a healthier society.

## Nudge theory in policy-making

Multiple treatments are always required to achieve sustainable change across populations. Public awareness campaigns that try to affect people’s behavior and the use of taxes and subsidies to change the affordability of various options. According to Albarracín et al. (15), providing information has, at most, a minor impact on behavior, even though each can contribute to efforts to influence behavior at scale.

Fiscal and economic interventions, on the other hand, have consistently larger effects (16, 17), but they are significantly less popular with citizens and, consequently, policymakers than information campaigns (18). Five of the seven most successful non-treatment interventions generally regarded as nudges in an analysis of 30 potential strategies for policymakers to reduce obesity in the UK by half by 2030 involved regulations to control food availability, placement, advertising, and labeling (19).

Over the past 10 years, the application of nudge theory (20) by governments has become more popular in a number of international jurisdictions. Richard Thaler and Cass Sunstein’s well-known book, Nudge—Improving Decisions about Health, Wealth, and Happiness, popularized the nudge theory, which has its roots in the development of behavioral science and psychology (21). The study and use of the nudge theory have exploded in the past 10 years across a wide range of fields, including public policy, design, political science, behavioral economics, and behavioral sciences.

A nudge is an indirect, frequently subtle intervention used to influence an individual’s or group’s decisions by positively reinforcing predictable, generally cost-effective, or innocuous behavior. However, the nudge principle is essential to the overall architecture of choice, as it gently modifies the limits of rationality, biases, and habits to affect an individual’s or a group’s judgment, decision, or choice in an expectable, frequently preset way (20).

Though many governments within the Organization for Economic Cooperation and Development (OECD) have endorsed nudge theory in one way or another, its application has never been impervious to criticism in democratic and scholarly discourse, despite the fact that its use has expanded among governments and policymakers. Nudge theory’s supporters see it as a helpful instrument for governments to perpetuate favorable results, but its detractors see it as a kind of (22), frequently hidden, government and policymaking.

However, the response to the query, “What kind of nudge works best?” relies on the local community’s history, culture, way of life, and customs in addition to its health problems. Furthermore, it is difficult to anticipate long-term impacts from nudges, even while short-term effects are possible. Therefore, a system that creates and consistently applies a variety of nudges based on community conditions is required to promote healthy aging.

A single division or even an individual may find it difficult to develop and apply different nudges. Health professionals, governments, and local communities should form alliances and exchange knowledge, experiences, and proficiency in order to provide essential services to those who need them (23).

Along with increasingly complex and varied administrative issues, many governments are confronted with severe budgetary constraints. The Western Pacific region in particular is experiencing severe fast aging (24). In order to create a healthy aging society, nudges—a cost-effective strategy for encouraging desired behaviors—have therefore gained international traction. Indeed, there has been much discussion about the use and application of behavioral economics to health policy in an aging society (25), and nudge has been included in older adults care (26).

## Ethics of nudging

The subject of whether governments or public organizations should be permitted to use nudge interventions to influence people’s behavior has generated a lot of ethical discussion. Some people find it disturbing and incompatible with individual liberty for governments to employ behavioral insights to influence citizens’ behavior (27). Others, like Peter John, contend that this anxiety is unwarranted and that rather than worrying about potential violations of autonomy, we should concentrate on creating sound public policies: “If they are adequately approved, supported by evidence, properly evaluated, and where any potential adverse impacts are outweighed by the benefits, so long as individual rights are not violated, policy makers can choose them [i.e., public policies]” (28). Some people share this opinion and contend that nudges are tools or instruments that do not require any particular explanation (29). Although there is much to support this viewpoint, if we examine particular government actions, the concept is refuted.

Roberts (30) asserts that the government bears an ethical duty to consider the distributional effects of nudges. The distributional consequences of salience nudges are not well understood, despite their significance. This may be partially because social scientists generally assume that knowledge has a positive effect on customers; more information is generally believed to help consumers make better selections based on their preferences.

Furthermore, policymakers frequently have to choose between competing obligations, and it must be evident how actions are justified by the fundamental values that the government is obligated to uphold (6). As long as the methods used are open and democratic, using nudges to influence people’s behavior is morally acceptable. Nudges should be evaluated ethically on an individual basis rather than in a generalized manner well (31).

## Intervention design and choice architecture

In recent years, behavioral science-informed intervention strategies have surfaced as a supplement to logical agent-based strategies. Their approach to decision-making acknowledges the limitations of people’s capacity for rational thought. Boundedly rational decision-makers are vulnerable to ostensibly irrational environmental influences, such as the presentation or structure of information, because they frequently build their preferences spontaneously based on cognitive shortcuts and biases (32).

People can influence decisions that better consider how individuals actually react to the circumstances in which they make them by developing a more thorough understanding of human decision-making, or “choice architecture” (21). They can make better decisions by using the same mistakes that trip them up (33).

At the same time that the shortcomings of conventional methods have become evident, policymakers have been interested in policies that alter the setting or “nudge” people in specific directions [see (34)]. Therefore, institutions, whether public or private, have taken on the role of “choice architects” whenever they are able to create sets of possibilities from which individuals can select. The state becomes a choice architect for its citizens’ health when it implements policies meant to influence the choices that its residents make about their well-being. Institutions whose primary focus is health (or health care) may not necessarily need to implement such policies. Even if they are centered in other state-run agencies like food, agriculture, or transportation, they may include programs that have an impact on health (35).

Moreover, a thorough assessment of the evidence supporting nudge (choice architecture) treatments is conducted by Hollands et al. (36). Though it does not exclude conscious and reflective activities, this definition particularly emphasizes the weight in nudge theory on automatic processes, which is why minimum conscious interaction is necessary. The physical and social aspects of microenvironments are also covered by this definition; that is, the emphasis is on the particular context, specifically interventions that entail making changes to small-scale physical and social environments (e.g., spaces such as workplaces) (37).

The idea that people will automatically select a “better” (i.e., healthier, more sustainable, more environmentally friendly, or more financially attractive) option once the right stimulus is found, is the promising part of choice architecture approaches (38). This will make costly enforcement structures redundant. The goal of choice architecture interventions based on decision information provision is to make decision-related information more accessible, understandable, and/or personally meaningful to the decision maker. Providing social reference information that lessens situational ambiguity and aids in overcoming ambiguity over proper behavioral responses is one approach to doing this.

Nonetheless, the context reliance is used by choice architecture interventions that center on modifications to the decision structure to affect behavior through the format of decision-making or the layout of choice alternatives. Choice default, or a predetermined selection of an alternative that prevails in the event that no active choice is made, is one of the most well-known instances of this intervention strategy (9).

But systematic studies examining the application of choice architecture approaches to bring about change at the micro, meso, and macro levels are still in their infancy, particularly when it comes to interventions at the meso and macro levels. Choice architecture and nudge have been utilized in a variety of scenarios since their inception, including a broad range of treatments, multiple behaviors, environmental contexts [such as physical activity (39), food intake (40, 41), or levels of analysis (41, 42)].

Nevertheless, nudge interventions can be developed using a variety of process-type frameworks. It is preferable for policymakers to use straightforward techniques when implementing nudges in order to aid stakeholders and beneficiaries in comprehending the idea of nudge intervention. The “three steps to design nudge” are the most straightforward process and were created using the OECD’s BASIC framework for behaviorally informed policymaking, which stands for behavior, analysis, strategies, intervention, and change (43). The first phase is to “identify target behavior,” and the second is to “use a behavior process map to establish the friction intervention and fuel of the behavior.” And finally, “Design and implement nudge” is the third phase (14).

## Nudge choice and libertarian paternalism

One’s adherence to nudge ethical values determines the response to the decision-making interrogation. Thaler and Sunstein claim that their method is paternalistic, but they also want to demonstrate that it is consistent with libertarian principles, which is why they call it “libertarian paternalism.” This designation is most likely due to the negative connotations associated with paternalism, particularly when it comes to health and medicine. The primary perception is that it denies people their freedom of choice.

Paternalism can also be seen as love and concern, from a father to his children or from the more powerful and educated to the less fortunate. Paternalists believe that certain individuals, such as doctors, public health authorities, choice architects, etc., are better able to determine what is best for individuals than the people themselves. They are obligated to assist others because they possess this information.

While liberalism often has respectable connotations, it can also be interpreted as a sign of carelessness and a refusal to assume responsibility for fellow people, in addition to guaranteeing freedom of choice and noninterference. As long as the interventions have a proven beneficial impact on the people being nudged, there should not be any objections to nudges if one supports paternalism. For those who view (paternalistic) care and consideration as beneficial, nudging may be referred to as gentle or mild paternalism (44).

Although nudges have been shown to be successful in increasing health without restricting people’s freedom (38), the paternalistic nature of the method has led to much criticism. A nudge transfers accountability to experts and those in positions of authority by assuming that people are not logical agents who are capable of making better choices (45). The phrase “libertarian paternalism,” however, is used by Thaler and Sunstein (46) to emphasize the freedom of choice while assigning some accountability to the nudger (i.e., the individual or group initiating the nudge).

The degree of intrusiveness of the nudge, its perceived effectiveness, and the goal of the choice architect all influence how widely these measures are accepted in different cultural contexts (e.g., scientists nudging hypothetical interventions are more reputable than governments recommending forth non-hypothetical ones) (47). Sunstein et al. (48) have demonstrated that countries with politically liberal environments have carried out the majority of nudging interventions.

Eighteen countries were included in national surveys conducted between 2015 and 2018. The main finding is that, although most people support health and safety initiatives, the world’s countries seem to fit into three different groups: The first set of countries like Canada, which are mostly liberal democracies, have sizable majorities that support nudges whenever they. The second group of countries like US have overwhelming majorities that support almost all nudges.

The third group of countries like Japan, typically exhibit majority approval of nudges, but at noticeably lower approval rates (49).

Nevertheless, by influencing consumption toward more sustainable and healthful food, size-based interventions may help shape health policies. Despite growing recognition of the structural causes of obesity, Smith and Toprakkiran’s (50) analysis of the development of the policy debate and measures surrounding obesity in the UK concluded that policy measures are mainly focused on choice, allowing the governments to avoid jeopardizing free markets and established economic interests. Five of the seven most successful non-treatment interventions found in an examination of 30 potential measures that policymakers may use to cut obesity in the UK in half by 2030 involved regulating food availability, placement, promotion, and labeling—generally referred to as nudges (19).

As the importance of policy packages has come to be understood more generally in recent years, nudge tactics—like those for smoking cessation and seat belt use—are increasingly being employed in conjunction with legal and fiscal mechanisms. As demonstrated by Huf et al. (51), text message reminders increase screening attendance. Effective cervical screening policies were created using the results of this experiment. There was a 4.8% increase in attendance, according to data released by Public Health England (52).

## Risks and safeguards

Free market advocates, have harshly contested this notion [e.g., (53)]. The fundamental problem is that nudging typically occurs without the full knowledge and control of decision makers, endangering their autonomy—that is, their ability to be the owners of their decisions. This creates problems since governments might not fully grasp what is best for people and may have their own hidden objectives and biases. Based on these lines of reasoning, this literature presents a compelling argument that government pressuring is inherently problematic and should be “escaped” (53).

However, this perspective shows that worries about autonomy and standards continue to dominate the ethical discussion surrounding healthcare nudges. Particularly in therapeutic and consent contexts, reflective nudges that encourage consideration received greater support than automatic nudges that covertly alter default behaviors. When quantifiable advantages to public health outweigh minimal interference, consequentialist reasoning supports nudges as long as transparency safeguards are maintained (54).

Therefore, although there is evidence to support the short-term effectiveness of nudges, it is problematic if people’ conscious decisions are questioned. It is a contentious danger even though not all nudges such as reminders or cautions target behavioral biases and do not replace policy-design but rather enhance or supplement it [e.g., (55)].

## Conclusion

In order to create a healthy society, nudging is a low-cost policy intervention strategy that may enhance and strengthen established laws and initiatives. It can be applied to encourage both people and societies to adopt positive habits. It can be used to improve living and social environments, eradicate prejudice and discrimination against minorities and vulnerable populations, improve access to medical and nursing care resources, and encourage healthy lifestyle choices like diet, exercise, and sleep.

In many nations, behavioral science nudges have been extensively used in the formulation and execution of public policies. Because nudge application does not always require expertise, it can be used in the work of individual government officials who are crucial to the front lines of health policy and service delivery. Nudges can be applied to many different sectors of public health policy and can also be used to increase strategic efficiency. They can be utilized, specifically, to lessen administrative costs and enhance beneficiaries’ access to a range of health programs.

The likelihood of behavioral change rises when nudges are combined with other strategies, including information sharing, financial incentives, and legislation. Therefore, adding nudges to health policy will hasten the implementation of policies that move society toward larger-scale health.

## Data Availability

The original contributions presented in the study are included in the article/supplementary material, further inquiries can be directed to the corresponding author.
